# Continuity in general practice and hospitalization patterns: an observational study

**DOI:** 10.1186/s12875-020-01361-0

**Published:** 2021-01-14

**Authors:** Michel Wensing, Joachim Szecsenyi, Gunter Laux

**Affiliations:** Department of General Practice and Health Services Research, University Hospital Heidelberg, Im Neuenheimer Feld 130.3, 69120 Heidelberg, Germany

**Keywords:** Continuity of care, Hospitalization patterns, General practice, Health services research

## Abstract

**Background:**

High continuity of care is a key feature of strong general practice. This study aimed to assess the effect of a programme for enhancing strong general practice care on the continuity of care in Germany. The second aim was to assess the effect of continuity of care on hospitalization patterns.

**Methods:**

We performed an observational study in Germany, involving patients who received a strong general practice care programme (*n*=1.037.075) and patients who did not receive this programme (*n*=723.127) in the year 2017. We extracted data from a health insurance database. The cohorts were compared with respect to three measures of continuity of care (Usual Provider Index, Herfindahl Index, and the Sequential Continuity Index), adjusted for patient characteristics. The effects of continuity in general practice on the rates of hospitalization, rehospitalization, and avoidable hospitalization were examined in multiple regression analyses.

**Results:**

Compared to the control cohort, continuity in general practice was higher in patients who received the programme (continuity measures were 12.47 to 23.76% higher, *P*< 0.0001). Higher continuity of care was independently associated with lowered risk of hospitalization, rehospitalization, and avoidable hospitalization (relative risk reductions between 2.45 and 9.74%, *P*< 0.0001). Higher age, female sex, higher morbidity (Charlson-index), and home-dwelling status (not nursing home) were associated with higher rates of hospitalization.

**Conclusion:**

Higher continuity of care may be one of the mechanisms underlying lower hospitalization rates in patients who received strong general practice care, but further research is needed to examine the causality underlying the associations.

**Supplementary Information:**

The online version contains supplementary material available at 10.1186/s12875-020-01361-0.

## Background

Continuity of care is a key feature of strong general practice, which has shown to have positive effects on health and healthcare [1]. A systematic review of observational studies found that higher continuity of care was associated with lower mortality rates [2]. Other research showed that a higher continuity of care was associated with lowered hospitalizations and lowered healthcare costs [3, 4]. Qualitative research showed that patients relate continuity of care to increased feelings of security and confidence [5]. Different mechanisms for the positive impact of continuity of care have been suggested. For instance, it may enhance physician inclination to provide pro-active health and enhance the responsiveness to emerging health problems [1, 2]. Most research on continuity of care is from a small number of countries, many of which have long established strong general practice care systems (e.g. United Kingdom, Denmark, and The Netherlands) as well as from a few other countries (United States, Canada). Our study concerned Germany, where the strength of general practice may be described as mixed, overall moderate, yet growing [6].

The conceptualization of continuity of care is challenging as it is not consistently defined and existing definitions overlap with other concepts, such as coordination, integration, and case management [7]. Continuity of care has been defined and measured in terms of patient-experienced continuity (‘the patient feels continuously known and cared for’), interpersonal continuity (‘the patient sees the same providers over time’), informational continuity (‘all healthcare providers always have relevant information on the patient’), and team continuity (‘team members remain the same and have all relevant information on the patient’) [7]. Haggerty et al. [8] distinguished between relational continuity (an ongoing therapeutic relationship between a patient and one or more providers), informational continuity (the use of information on past evens and personal circumstances to make current care appropriate for each individual), and management continuity (a consistent and coherent approach to the management of a health condition that is responsive to a patient’s changing needs). In the present study, we focused on relational continuity and used factual measurements, using a large data-base of claims data.

In previous decade, health system reforms to strengthen general practice care (‘Hausarztzentrierte Versorgung’) have been implemented in various states in Germany [9, 10]. These reforms reinforced the general practitioner as the first point of access to healthcare for most adults and most health problems. This change has been effectively implemented in Baden-Württemberg, a region with about 11 million inhabitants in South Germany [9]. A previous study showed that this probably reduced the number of hospital admissions and the mortality in patients who received the programme [11, 12]. The impact of the programme on continuity in general practice had not yet been examined. Previous studies found a higher number of contacts in general practice in patients who received the programme [9, 10], which may be associated with either lowered or increased continuity.

High continuity in general practice may lower hospitalization rates, if it provides services that would otherwise require hospital admission or reduces the risk adverse events that require hospitalization. Hospitalizations have been the topic of much research and many intervention programmes [13]. The likelihood of admission to hospital is influenced by many factors beyond the nature of the health problem, including the capacity and quality of primary care, the presence of social support and other sources of community care. Nevertheless, having a regular source of primary care was found to be one of the few factors that consistently reduced hospitalization rates in diabetes patients [14]. The introduction of a named GP in primary care for patients aged 75 years and older did not change the continuity of care and was associated with higher numbers of hospitalizations [15]. It remains therefore uncertain whether high continuity of primary care actually lowers hospitalization rates.

The presented study had two specific objectives. The first was to assess the impact of the programme for strengthening general practice on the continuity of care. Second, it aimed to explore the effects of continuity in general practice on hospitalization patterns, independent of receiving the programme.

## Methods

### Design

This observational study was based on data, which are quarterly collected by a large health insurer for reimbursement of healthcare providers. The insurer (AOK Baden-Württemberg, Germany) covers about 45% of the population of about 11 million inhabitants in Baden-Württemberg. The data in this study refer to the year 2017 (1 January until 31 December).

### Study population

As in previous studies of strong general practice care [9, 11, 12], we included patients if they were adults (aged 18 years or older), lived in the region, had one or more visits to a physician in the region, were insured with AOK (without interruptions in the observed year), and were not registered with other healthcare contracts. Patients in the programme had a known connection to a specific general practitioner, because they provided informed consent before participation in the study. We compared them with all other eligible patients in the observed year, who are not registered in the programme. In the analysis, we linked these non-enrolled patients to the physician, whom they had visited in half or more of their ambulatory care contacts. If no such linkage was possible, they were excluded from analysis. We assumed that the most contacted physician was the general practitioner. A total of 212,203 out of 2,818,295 potential control patients (7.5%) were excluded.

### Programme

The programme for enhancing strong general practice care is specified in Paragraph 73b of the German Social Code Book Five. The general practitioner (GP) receives 40% additional reimbursement for included patients. The included patients did not have direct financial benefit. Physicians and patients can freely choose whether they enroll in the programme. The following requirements were specified:

- Prompt access to care. The practice organization of the physician should have specific features, such as daily consultation hours and a specific information technology infrastructure. Patients should experience shorter waiting times and absence of out-of-pocket payments for medication.
- Comprehensive medical care. The physician should regularly participate in continuing training in primary care-relevant domains.
- Computerized decision support. The physician should integrated decision support for medication prescribing in the daily routines.
- Disease management. The physician should participate in disease management programmes for diabetes, asthma/COPD, and coronary heart disease.
- Gate-keeping to secondary care. Referrals to specialist care should go through primary care, which implies that patients should not attend specialist care without referrals.
- Healthcare coordination. Specific requirements for information and communication between general practitioners and medical specialists were specified.
- Data-driven quality improvement. The physician should attend small-group quality improvement with feedback on their medication prescribing (‘quality circles’).

### Measures

All measures were based on health insurance claims data. We operationalized continuity of care in three coefficients, following previous research [16]: the Usual Provider of Care (UPC), the Herfindahl Index (HI), and the Sequential Continuity Index (SCN) (Table 1). We did not use the Bice-Boxerman Continuity of Care Index [16], because it is similar to the Herfindahl Index but would imply a restriction of the data-set. For all three coefficients applies that higher values indicate higher continuity of care. UPC indicates the concentration of contacts with a specific healthcare provider within an episode of care. HI indicates the concentration of contacts within providers across all providers involved in care for a patient. SCN indicates sequential contacts with the same provider for all providers aggregated.

**Table 1:** Claims based coordination measures

Measure	Formula	Concept
Usual Provider of Care (UPC)	$\max \left(\frac{n_i}{n}\right)$	Concentration of care with a single provider
Herfindahl-Index (HI)	${\sum}_{i=1}^P{\left(\frac{n_i}{n}\right)}^2$	Degree of coordination required between different providers during an episode
Sequential Continuity Index (SECON)	$\frac{\sum_{j=1}^{n-1}{c}_j}{n-1}$	Number of handoffs of information required between providers (only values if 2 or more visits)

**Formula variables**

*n*: total number of visits during episode

*n*_*i*_: number of visits to provider *P*_*i*_

*P*: total number of providers

*c*_*j:*_ indicator of sequential visits to same provider*c*_*j*_ = 1, if visits *j* and *j+ 1* are to the same provider, *c*_*j*_ = 0 otherwise

Hospitalizations were operationalized as in previous research of GP-centred care [3] in terms of total hospitalizations (number of hospitalizations per patient), rehospitalizations within 4 weeks (independent of admission diagnoses), and potentially avoidable hospitalizations. The latter was determined on the basis of “ambulatory care-sensitive conditions”, which – based on ICD-10 codes – include the most prevalent conditions for hospitalizations that could have potentially been handled in ambulatory care. The underlying calculus is described in detail elsewhere [17] and summarized in Supplement 1. The three measures are based on count data per patient. The corresponding events may occur not (0), once (1) or more than once (*n*> 1) in a patient in the observed period.

We also extracted patient age (in years), sex (male, female), nationality (German, other), health insurance (member, family, retired), comorbidity (Charlson Index [18]), living in a nursing home, number of contacts in general practice care, and (for the intervention group) length of participation in the programme (in quarter years) from the data-base.

### Data-analysis

We calculated descriptive characteristics of patients (as listed above) for patients in the strong general practice care programme (intervention cohort) and patients not in the programme (control cohort). To address the research aims, we used linear mixed models [19] with GEEs (Generalized Estimating Equations) in combination with the Poisson distribution. In all regression models, the patient characteristics listed above, except for number of consultations and time in the programme, were included for correction of differences in composition of the two cohorts. Clustering of patients in GPs and GPs in practices was included in the models. For technical reasons, we had to exclude the patients who dropped out of the programme, because we could not assign those patients to one of the both groups. This drop-out rate was 1,4% in this cohort. The intervention and control patients had a one-year death rate of 0.0173 and 0.0178, respectively. Therefore, we did not consider loss to follow-up due to mortality in the analysis.

For the first research objective, concerning the effect of the programme on continuity in general practice, we compared the three coefficients of continuity of care between the cohorts. For the second research objective, concerning the effect of continuity on hospitalizations, we compared the cohorts with respect to rates of hospitalization, rehospitalization, and avoidable hospitalization and included the continuity of care coefficients as potential predictors (three separate models for each continuity coefficient). In this way, we examined the independent effect of continuity on hospitalizations (i.e. independent of receiving the programme).

We assessed models’ “goodness of fit” by the quasi-likelihood under the independence model criterion [20] and models with the best goodness of fit were preferred. We considered *P*-values smaller than 0.01 significant. Data storage and extraction was performed with MySQL Community Server × 64 (Oracle Corporation, Redwood Shores, CA, USA). Statistical analyses were performed using SAS 9.4 (SAS Institute Inc., Cary, North Carolina, USA).

## Results

Table 2 describes the characteristics of patients in the strong general practice care programme and the control cohort. In the programme, the mean age of patients was 57.2 years, 55.9% was female, and the Charlson score for comorbidity was 1.48 on average. The control cohort was slightly younger (54.0 years) and had less comorbidity (Charlson score: 1.12). As could be expected, patients in the programme had more contacts in general practice (mean: 13.56) than the control patients (mean: 8.86). Crude hospitalization rates were slightly higher in the strong general practice care programme than in control patients (mean per 100 patients: 28.7 versus 28.3).

**Table 2:** Description of patient population

	Intervention cohort	Control cohort	P-Value
Absolute numbers	1,037,075	723,127	–
Mean age (SD)	57.2 ± 18.8	54.0 ± 19.8	< 0.0001
Sex %women	55.9%	55.8%	n.s.
Nationality -%German	82.9%	79.8%	< 0.0001
Insurance			
%Member	53.6%	54.4%	< 0.0001
%Family	12.5%	14.7%	
%Retired	33.9%	30.9%	
Comorbidity Mean Charlson Index (SD)	1.48 ± 2.13	1.12 ± 1.85	< 0.0001
Patients living in nursing homes (n; %)	8628; 0.83%	8825; 1.22%	< 0.0001
Mean number of contacts in general practice care (SD)	13.56 ± 11.70	8.86 ± 11.06	< 0.0001
Hospital admissions per 100 patients (SD)	28.7 ± 78.6	28.3 ± 77.7	0.0003
Rehospitalizations in 4 weeks per 100 patients (SD)	22.8 ± 70.5	23.3 ± 69.9	< 0.0001
Potentially avoidable hospitalizations per 100 patients (SD)	15.8 ± 33.4	15.7 ± 33.5	0.0319
Mean number of quarter years in strong general practice care programme (SD)	25.9 ± 9.9	–	–

The scores for continuity of care coefficients in the two cohorts are presented in Tables 3 and 4. Continuity of care was overall high. For instance, the Usual Provider Index had a mean of 0.874 in control cohort. For patients in the strong general practice care programme, scores were 12.47 to 23.76% higher than in the control cohort (all differences statistically significant). The difference was highest for the Sequential Continuity Index, on which higher scores indicates the absence of transfers between different healthcare providers within an episode of care.

**Table 3:** Continuity of care in intervention and control cohorts

	Intervention cohort	Control cohort	Adjusted difference (SEM, P-value)
**Usual Provider Index** **(mean, SD)**	0.942 ± 0,103	0.874 ± 0.164	+ 12.47% (0.0007; < 0.0001)
**Herfindahl Index** **(mean, SD)**	0.909 ± 0.149	0.824 ± 0.213	+ 15.29% (0.0009; < 0.0001)
**Sequential Continuity Index (mean, SD)**	0.895 ± 0.178	0.789 ± 0.262	+ 23.76% (0.0013; < 0.0001)

**Table 4:** Predictors of continuity of care

	Estimation	95%-CI	SEM, P-Value
**Usual Provider Index**			
Programme (yes)	+ 12.47%	[+ 12.32%, + 12.61%]	0.0007, < 0.0001
Sex (male)	+ 0.690%	[+ 0.656%, + 0.726%]	0.0002, < 0.0001
Age	+ 0.070%	[+ 0.069%, + 0.071%]	0.0001, < 0.0001
Charlson-index score	−0.675%	[−0.686%, − 0.664%]	0.0001, < 0.0001
Nationality (German)	+ 0.134%	[+ 0.085%, + 0.183%]	0.0003, < 0.0001
**Herfindahl Index**			
Programme (yes)	+ 15.29%	[+ 15.10%, + 15.49%]	0.0009, < 0.0001
Sex (male)	+ 1.074%	[+ 1.026%, + 1.122%]	0.0002, < 0.0001
Age	+ 0.090%	[+ 0.088%, + 0.092%]	0.0001, < 0.0001
Charlson-index score	−1.038%	[− 1.053%, − 1.023%]	0.0001, < 0.0001
Nationality (German)	+ 0.211%	[+ 0.144%, + 0.277%]	0.0003, < 0.0001
**Sequential Continuity Index**			
Programme (yes)	+ 23.76%	[+ 23.50%, + 24.02%]	0.0013, < 0.0001
Sex (male)	+ 0.332%	[+ 0.272%, + 0.392%]	0.0003, < 0.0001
Age	+ 0.178%	[+ 0.176%, + 0.180%]	0.0001, < 0.0001
Charlson-index score	−0.683%	[− 0.665%, − 0.702%]	0.0001, < 0.0001
DMP participation (yes)	+ 0.562%	[+ 0.471%, + 0.654%]	0.0005, < 0.0001
Nationality (German)	+ 0.414%	[+ 0.334%, + 0.499%]	0.0004, < 0.0001

The expected lowering effect of continuity in general practice on hospitalization rates was confirmed for all continuity of care coefficients and all hospitalization outcomes (Tables 5, 6, 7). The largest effects were found for the Usual Provider Index and the Herfindahl Index on the overall hospitalization rates (9.74 and 8.76% relative reductions for a 10% absolute increase of continuity of care, respectively). The effect of a 10% increase on the continuity of care on hospitalization was larger than the effect of receiving the programme, which nevertheless remained statistically significant in 6 of 9 of the analyses.

**Table 5:** Predictors of overall Hospitalizations

	Estimation	95%-CI	SEM, P-Value
**Usual Provider Index**^**1**^			
**UPC**	**−9.74%**	**[−9.94%, − 9.54%]**	**0.0010, < 0.0001**
Programme (yes)	−1.16%	[−0.52%, − 1,79%]	0.0013, 0.0004
Sex (male)	−5.16%	[− 5.72%, −4.59%]	0.0029, < 0.0001
Age	+ 0.31%	[+ 0.29%, + 0.33%]	0.0001, < 0.0001
Charlson-index score	+ 26.48%	[+ 26.38%, + 26.59%]	0.0005, < 0.0001
Living in Nursing home (yes)	−6.94%	[−8.93%, − 4.96%]	0.0101, < 0.0001
**Herfindahl Index**^**2**^**:**			
**HI**	**−8.76%**	**[−9.06%, − 8.46%]**	**0.0015, < 0.0001**
Programme (yes)	−0.22%	[−1.69%, + 1,25%]	0.0075, n.s.
Sex (male)	−4.90%	[−5.77%, − 4.03%]	0.0045, < 0.0001
Age	+ 0.33%	[+ 0.29%, + 0.36%]	0.0002, < 0.0001
Charlson-index score	+ 26.26%	[+ 26.06%, + 26.47%]	0.0011, < 0.0001
Living in Nursing home (yes)	−7.05%	[−10.00%, − 4.10%]	0.0150, < 0.0001
**Sequential Continuity Index**^**3**^			
**SECON**	**−3.52%**	**[− 3.66%, − 3.38%]**	**0.0007, < 0.0001**
Programme (yes)	−6.34%	[− 6.98%, − 5,70%]	0.0075, n.s.
Sex (male)	−1.80%	[− 2.37%, − 1.23%]	0.0029, < 0.0001
Age	+ 0.27%	[+ 0.25%, + 0.29%]	0.0001, < 0.0001
Charlson-index score	+ 29.98%	[+ 29.26%, + 30.69%]	0.0037, < 0.0001
Living in Nursing home (yes)	−7.35%	[−9.34%, −5.36%]	0.0101, < 0.0001

**Legend:** 1. Reduction in the amount of 2.78 hospitalizations per 100 patients. 2. Reduction in the amount of 2.50 hospitalizations per 100 patients**.** 3. Reduction in the amount of 1.01 hospitalizations per 100 patients

**Table 6:** Predictors of rehospitalizations within 4 weeks

	Estimation	95%-CI	SEM, P-Value
**Usual Provider Index**^**1**^**:**			
**UPC**	**− 5.33%**	**[− 5.87%, − 4.80%]**	**0.0027, < 0.0001**
Programme (yes)	−1.66%	[− 3.34%, + 0,02%]	0.0086, 0.053
Sex (male)	− 2.53%	[−4.02%, − 1.04%]	0.0076, < 0.0001
Age	− 1.46%	[− 1.50%, − 1.41%]	0.0002, < 0.0001
Charlson-index score	+ 23.55%	[+ 23.30%, + 23.80%]	0.0013, < 0.0001
Living in Nursing home (yes)	− 18.16%	[− 23.25%, − 13.07%]	0.0260, < 0.0001
**Herfindahl Index**^**2**^**:**			
**HI**	**− 4.71%**	**[−5.11%, − 4.31%]**	**0.0020, < 0.0001**
Programme (yes)	− 0.96%	[− 2.63%, + 0,72%]	0.0085, n.s
Sex (male)	−2.46%	[−3.96%, − 0.97%]	0.0076, < 0.0012
Age	− 1.45%	[− 1.50%, − 1.40%]	0.0002, < 0.0001
Charlson-index score	+ 23.48%	[+ 23.23%, + 23.73%]	0.0013, < 0.0001
Living in Nursing home (yes)	− 18.17%	[− 23.26%, − 13.08%]	0.0260, < 0.0001
**Sequential Continuity Index**^**3**^**:**			
**SECON**	**− 2.16%**	**[− 2.55%, − 1.77%]**	**0.0020, < 0.0001**
Programme (yes)	−4.15%	[−5.85%, − 2,46%]	0.0086, < 0.0001
Sex (male)	−0.77%	[− 2.27%, + 0.74%]	0.0077, n.s.
Age	−1.39%	[− 1.44%, − 1.34%]	0.0003, < 0.0001
Charlson-index score	+ 23.71%	[+ 23.46%, + 23.96%]	0.0013, < 0.0001
Living in Nursing home (yes)	− 18.82%	[−23.92%, − 13.72%]	0.0260, < 0.0001

**Legend.** 1. Reduction in the amount of 1.23 rehospitalizations per 100 patients. 2. Reduction in the amount of 1.08 rehospitalizations per 100 patients. 3. Reduction in the amount of 0.49 rehospitalizations per 100 patients

**Table 7:** Predictors of potentially avoidable hospitalizations

	Estimation	95%-CI	SEM, P-Value
**Usual Provider Index**^**1**^**:**			
**UPC**	**− 3.83%**	**[− 4.92%, − 3.24%]**	**0.0030, < 0.0001**
Programme (yes)	−2.32%	[− 4.23%, − 0.42%]	0.0097, 0.0173
Sex (male)	+ 9.00%	[+ 7.47%, + 10.53%]	0.0078, < 0.0001
Age	+ 1.45%	[+ 1.39%, + 1.51%]	0.0003, < 0.0001
Charlson-index score	+ 1.86%	[+ 1.59%, + 2.13%]	0.0014, < 0.0001
Living in Nursing home (yes)	+ 14.67%	[+ 11.16%, + 18.18%]	0.0179, < 0.0001
**Herfindahl Index**^**2**^**:**			
**HI**	**− 3.02%**	**[− 3.46%, −2.58%]**	**0.0023, < 0.0001**
Programme (yes)	−2.22%	[−4.13%, − 0.32%]	0.0097, 0.0222
Sex (male)	+ 9.03%	[+ 7.50%, + 10.56%]	0.0078, < 0.0001
Age	+ 1.45%	[+ 1.40%, + 1.51%]	0.0003, < 0.0001
Charlson-index score	+ 1.83%	[+ 1.56%, + 2.10%]	0.0014, < 0.0001
Living in Nursing home (yes)	+ 14.62%	[+ 11.11%, + 18.13%]	0.0179, < 0.0001
**Sequential Continuity Index**^**3**^**:**			
**SECON**	**−2.45%**	**[− 2.87%, − 2.02%]**	**0.0022, < 0.0001**
Programme (yes)	−3.16%	[−5.07%, −1.26%]	0.0097, 0.0011
Sex (male)	+ 7.69%	[+ 6.16%, + 9.21%]	0.0078, < 0.0001
Age	+ 1.41%	[+ 1.35%, + 1.46%]	0.0003, < 0.0001
Charlson-index score	+ 1.97%	[+ 1.70%, + 2.24%]	0.0014, < 0.0001
Living in Nursing home (yes)	+ 14.49%	[+ 10.98%, + 18.00%]	0.0179, < 0.0001

**Legend.** 1. Reduction in the amount of 0.60 potentially avoidable hospitalizations per 100 patients. 2. Reduction in the amount of 0.48 potentially avoidable hospitalizations per 100 patients. 3. Reduction in the amount of 0.39 potentially avoidable hospitalizations per 100 patients

The regression analysis showed that higher age, female sex, higher morbidity (Charlson-index), and home-dwelling status (not nursing home) were associated with higher risk of hospitalization. For potentially avoidable hospitalization, however, male sex and living in a nursing home were associated with higher hospitalization risk (Tables 5, 6, 7).

## Discussion

### Summary

Patients in the strong general practice care programme had higher continuity in general practice than a comparable cohort of control patients. This means that they more often attended the same physician and had fewer handoffs. High continuity in general practice was associated with lowered hospitalization rates, independent of receiving the programme. Given the cross-sectional nature of the study, we cannot conclude that high continuity in general practice caused lowered hospitalization rates, even though this seems a plausible explanation.

### Strengths and limitations

The strengths of the study are the naturalistic setting, the high validity of the claims data, and the large-scale and sustained programme of strong general practice care. The health insurance claims data cover almost half of the population in the state, but specific groups (e.g. people with high income) are less well represented. Patients in Germany do not necessarily have a regular GP, because they have free choice of physician. In the broader evaluation of the programme for strong general practice care, it was decided to compose a control cohort of patients who had at least some continuity of physician. This might have led to an overestimation of the overall continuity of care in the control cohort and an underestimation of the beneficial effects of the programme, as medical care in the control cohort had higher continuity than average. Thus, in reality the effect of the programme on continuity in general practice may be larger than found in our study. Given the methods used for linking patients to physicians, we are fully confident that patients in the intervention cohort have been linked to a specific GP (regardless of whether this was the most frequently attended physician). The programme for strengthening general practice was only open for GPs and enrolment required an active decision, including written informed consent. In the control cohort, the method guaranteed that patients were linked to a GP as the medical discipline was recorded for all physicians and only GPs were included in the analysis.

### Interpretation of findings

As a background for understanding the findings of the study, some aspects of German healthcare need to be highlighted. As compared to countries that dominate the international research literature on primary care (e.g. United Kingdom, Denmark, Netherlands), the number of visits of patients to GPs is high in Germany. This is probably related to the reimbursement system, which relates payment to patient attendance per quarter of year and reimburses specific services separately. Furthermore, the number of hospital beds and the probability of hospitalisation is higher in Germany than in many other countries. This may provide an easy starting point for reduction of hospitalization.

Looking at other studies that used similar measures of relational continuity in general practice, we can conclude that the continuity in general practice is high in the observed cohorts. For instance, a study of general practice in England showed an average Usual Provider Continuity of 0.61 overall, albeit a little higher (0.70) in small practices with up to three physicians [3]. This index was 0.87 in the control cohort in our study, and higher in the strong general practice care programme. A study of general practice care in the United States showed a Usual Provider Continuity of about 0.80 on average [4], which is closer to our study but still lower. For a good interpretation of the findings of this study, it should be noted that the average number of visits of a patient to a GP is estimated on 10 to 15 per year, which is substantially higher than in many other countries.

Regardless of the absolute degree of the continuity of care, its lowering effect on hospitalization rates was found across the range of observed values across studies (e.g. Usual Provider Continuity values from 0.50 to 1.00). The effect of continuity on hospitalization in our study seemed even higher than in other studies. The English study looked at avoidable hospital admissions and found 3.87% lower rates in the tertile of high continuity as compared to the tertile of medium continuity [3]. The effect is (about three times) smaller than the effect in our study (as the relative risk reductions in Tables 3 and 4 refer to change of one decile on the continuity of care measure). The U.S. study reported about 7% lowering of total hospitalization rates, related to quintiles on the continuity of care measure [4]. This effect is (almost three times) smaller than the effect found in our study.

We can only speculate about the reasons for the stronger association between continuity of general practice and hospitalization rates in Germany, which is particularly striking as the absolute degree of continuity of care was already very high. The programme had a number of components that may have contributed to the lowering of hospitalization rates, such as the structured management of chronic diseases [11]. Also, the utilization of hospitals is relatively high in Germany compared to other countries. For instance, the Organisation for Economic Collaboration and Development Health Database reports 25.5 hospital discharges per 100 inhabitants in 2017 for Germany (around the 28.7 hospital admissions per 100 inhabitants in our study). The numbers of hospital discharges per 100 inhabitants are, for instance, 18.6 for France, 17.1 for Switzerland, 12.3 for the United Kingdom, and 9.2 for The Netherlands [21]. Although these numbers do not convey information on the appropriateness of hospital care, they may suggest a potential to reduce the number of hospital admissions in Germany. We assumed that lowered rates of hospitalizations are beneficial, not undesired delay of hospital admission, because of the wide availability and easy access to hospital care in most of South Germany.

In Germany, patients in nursing homes are medically managed by primary care physicians. If all other things are equal, we found that patients in nursing homes have a lowered probability for hospitalisation. It may be possible that the treatment and care for these patients is, on average, better than for patients at home. However, the number of patients in nursing homes was low, so we do not want to interpret this finding too prominently.

### Implications for research and practice

Future research should sort out the causality of the identified associations. For instance, the findings might be explained by a reversed causal explanation: lowered continuity in general practice (i.e. seeing more different physicians) after hospital admissions. Future analyses should model the timing of hospitalizations in relation to the experienced degree of continuity in general practice. This would also help to provide insight into the role of continuity of care in the causal chain underlying the lowering impact of the programme for strengthening general practice on hospitalizations. Randomized trials provide the strongest evidence for causality, but these may not be feasible for large-scale study in a naturalistic setting.

Future studies may explore whether suboptimal interpersonal continuity of care can be compensated for by information continuity and team continuity, which seems unavoidable in the context of current physician shortages and physicians’ perceived needs for work-life balance. In Germany, many general practice care practices are run by one or two physicians, although there is a trend towards practices with more staff (both physicians and assistants) [22]. This trend is present in other countries, such as the United Kingdom where continuity of general practice is lower than in Germany, particularly in large general practice care practices [3]. While this trend may be unavoidable, its potential impact on the quality and outcomes of healthcare needs attention.

## Conclusions

This study measured continuity of care on the basis of an administrative database of a large health insurer in South Germany. The study suggested that higher continuity of care may result in lower hospitalization rates in patients who received strong general practice care. Further research is needed to examine the causality underlying the associations.

## Supplementary Information

**Additional file 1 Table S1.** List of ICD 10-GM Codes Used to Identify Hospitalizations for Ambulatory Care–Sensitive Conditions.

## Data Availability

The datasets used and analysed during the current study available from the AOK Baden-Wuerttemberg on reasonable request.

## Abbreviations

AOK: Allgemeine Orts Krankenkasse (‘Regional Sickness Fund‘)
COPD: Chronic Obstructive Pulmonary Disease
GEEs: Generalized Estimating Equations
GP: General practitioner
HI: Herfindahl Index
ICD-10: International Classification of Diseases, version 10
UPC: Usual Provider of Care
SCH: Sequential Continuity Index
