# Supplementary material for: Continuity in general practice and hospitalization patterns: an observational study
Source: BMC Fam Pract. 2021 Jan 14;22:21. doi: 10.1186/s12875-020-01361-0 (PMC7809859; doi:10.1186/s12875-020-01361-0)
Supplement: Supplementary file 1 — Additional file 1 Table S1. List of ICD 10-GM Codes Used to Identify Hospitalizations for Ambulatory Care–Sensitive Conditions. [file 12875_2020_1361_MOESM1_ESM.pdf]

Supplement 1

**Supplemental Table 1. List of ICD 10-GM Codes Used to Identify Hospitalizations for Ambulatory Care–Sensitive Conditions**	
Ambulatory Care Sensitive Condition	***ICD10-GM Code***
Angina	I20, I24.0, I24.8, I24.9
Asthma	J45, J46
Cellulitis	L03, L04, L08.0, L08.8, L08.9, L88, L98.0
Congestive heart failure	I11.0, I50, J81
Convulsion and epilepsy	G40, G41, R56, O15
Chronic obstructive pulmonary disease	J20, J41, J42, J43, J47
Dehydration and gastroenteritis	E86, K52.2, K52.8, K52.9
Dental conditions	A69.0, K02, K03, K04, K05, K06, K08, K09.8, K09.9, K12, K13
Diabetes complications	E10.0–E10.8, E11.0–E11.8, E12.0–E12.8, E13.0–E13.8, E14.0– E14.8
Ear, nose and throat infections	H66, H67, J02, J03, J06, J31.2
Gangrene	R02
Hypertension	I10, I11.9
Influenza and pneumonia	J10, J11, J13, J14, J15.3, J15.4, J15.7, J15.9, J16.8, J18.1, J18
Iron-deficiency anemia	D50.1, D50.8, D50.9
Nutritional deficiency	E40, E41, E42, E43, E55.0, E64.3
Other vaccine preventable diseases	A35, A36, A37, A80, B05, B06, B16.1, B16.9, B18.0, B18.1, B26, G00.0, M01.4
Pelvic inflammatory disease	N70, N73, N74
Perforated/bleeding ulcer	K25.0–K25.2, K25.4–K25.6, K26.0–K26.2, K26.4–K26.6, K27.0–K27.2, K27.4–K27.6, K280–282, K284–K286
Pyelonephritis	N10, N11, N12, N13.6
Alcohol-related diseases	F10
Atrial fibrillation and flutter	I47.1, I47.9, I49.5, I49.8, I49.9, R00.0, R002, R00.8
Constipation	K59.0
Fractured proximal femur	S72.0, S72.1, S72.2
Dyspepsia and other stomach function disorders	K30, K21
Hypokalemia	E87.6
Migraine/acute headache	G43, G44.0, G44.1, G44.3, G44.4, G44.8, R51x
ICD-10-GM = International Classification of Diseases-, 10th Revision – German Modification.	
